# Significant Changes in Low-Abundance Protein Content Detected by Proteomic Analysis of Urine from Patients with Renal Stones After Extracorporeal Shock Wave Lithotripsy

**DOI:** 10.3390/biology14050482

**Published:** 2025-04-27

**Authors:** Elena Carestia, Fabrizio Di Giuseppe, Mohammad Kazemi, Massoumeh Ramahi, Uditanshu Priyadarshi, Patricia Giuliani, Piergustavo De Francesco, Luigi Schips, Carmine Di Ilio, Renata Ciccarelli, Patrizia Di Iorio, Stefania Angelucci

**Affiliations:** 1 Center for Advanced Studies and Technologies (CAST), University “G. d’Annunzio” of Chieti-Pescara, Via Luigi Polacchi 13, 66100 Chieti, Italy; carestiaelena@gmail.com (E.C.); fabrizio.digiuseppe@unich.it (F.D.G.); mohammad.kazemi@phd.unich.it (M.K.); massoumeh.ramahi@phd.unich.it (M.R.); uditanshu.priyadarshi@phd.unich.it (U.P.); carmine.diilio@unich.it (C.D.I.); stefania.angelucci@unich.it (S.A.); 2 Department of Sciences, ‘G d’Annunzio’ University of Chieti-Pescara, Via Vestini 31, 66100 Chieti, Italy; 3 Department of Innovative Technologies in Medicine and Dentistry, University “G. d’Annunzio” of Chieti-Pescara, Via dei Vestini 31, 66100 Chieti, Italy; 4 Department of Aging Medicine and Sciences (DMSI), ‘G d’Annunzio’ University of Chieti-Pescara, Via Vestini 31, 66100 Chieti, Italy; 5 Department of Medical, Oral and Biotechnological Sciences, ‘G d’Annunzio’ University of Chieti-Pescara, Via Vestini 31, 66100 Chieti, Italy; patricia.giuliani@unich.it (P.G.); luigi.schips@unich.it (L.S.); patrizia.diiorio@unich.it (P.D.I.); 6 Urology Unit, Azienda Sanitaria Locale 2, San Pio Hospital, Via San Camillo de Lellis, 66054 Vasto, Italy; piergustavodf@hotmail.it

**Keywords:** extracorporeal shock wave lithotripsy (ESWL), patients’ urine, bidimensional electrophoresis (2DE), mass spectrometry analysis

## Abstract

Extracorporeal shock wave lithotripsy (ESWL), although a highly effective method for the treatment of kidney stones, can cause significant kidney damage. Since urinary protein composition directly reflects kidney function, proteomic analysis of this fluid may be useful to identify changes in protein levels induced by patient exposure to ESWL as a sign of kidney damage. To this end, we collected urine samples from 80 patients with nephrolithiasis 2 h before and 24 h after exposure to ESWL, which were concentrated and subsequently processed with a commercially available enrichment method to extract low-abundance urinary proteins. These were then separated by 2D electrophoresis and subsequently analyzed by a proteomic approach. A large number of proteins were identified as being related to inflammatory, fibrotic, and antioxidant processes and changes in the levels of some of them were confirmed by Western blot analysis. Therefore, although further experimental confirmation is needed, our results demonstrate that ESWL significantly influences the low urinary protein profile of patients with nephrolithiasis. Notably, among the identified proteins, matrix metalloproteinase 7, alpha1-antitrypsin, and clusterin, as well as dimethyl arginine dimethyl amino hydrolase 2 and ab-hydrolase, may play an important role as putative biomarkers in the monitoring and management of ESWL-induced renal damage.

## Simple Summary

Although extracorporeal shock wave lithotripsy (ESWL) is effectively used to remove kidney stones of <2–2.5 cm in size, it can also cause kidney damage. Since urine directly reflects renal functions, it can be used to find potential indicators of possible kidney dysfunctions. In particular, this can be achieved by analyzing the protein composition of urine. In this work, using a more advanced technology known as mass spectrometry, we examined urine collected from patients with nephrolithiasis 2 h before and 24 h after their treatment with ESWL. After concentrating urine samples to increase their protein content, our proteomic analysis showed significant changes in a number of low-abundance urinary proteins that are involved in tissue inflammation and fibrosis, as well as oxidative processes. Notably, some of them could be considered as bioindicators of kidney damage caused by ESWL, thus facilitating diagnosis and therapeutic treatments.

## 1. Introduction

Extracorporeal shock wave lithotripsy (ESWL) is the preferred and relatively non-invasive method for removing renal stones smaller than 2–2.5 cm. Although it is well tolerated by patients and has a high success rate, concerns about its safety have arisen since its introduction in 1980. Indeed, several reports indicate morphological and functional changes in the kidney after ESWL [1,2,3]. In particular, traumatic injury can be induced by the wave physical force, while ischemic/hypoxic renal damage is caused by renal vasoconstriction associated with tissue bleeding. Accordingly, this series of events can lead to acute kidney injury (AKI) [4,5]. For a long time, AKI has been diagnosed by measuring serum creatinine levels and more recently also by serum levels of plasma cystatin C and C-reactive protein [6]. However, there is an increasing need to search for more sensitive biomarkers to detect post-ESWL renal injury [7].

Since the protein composition in urine directly reflects kidney functions, the use of urinary proteome may represent a powerful tool to identify possible biomarkers expressed during pathophysiological processes affecting the kidneys [8,9,10]. Therefore, potential urinary biomarkers for the sensitive and specific detection of human AKI [11] or the diagnosis of calcium-oxalate nephrolithiasis have been investigated [12]. Urine proteome studies in former stone patients and stone-free controls have also been performed, leading to the identification of a number of proteins in the stone group that may serve as potential useful candidates for the diagnosis and treatment of urolithiasis [13]. Unfortunately, reports regarding the pattern of human urinary proteins after ESWL exposure in nephrolithiasis patients are still modest. For example, some studies have reported that urinary levels of N-acetyl beta-glucosaminidase (NAG) and beta-galactosidase, as well as alpha1- and alpha2-macroglobulin, increase significantly after lithotripsy, suggesting that these proteins may be indicative of renal damage occurring after treatment, as reviewed in [14]. However, the presence of highly abundant proteins in some biological fluids, including urine, may make it difficult to detect disease-associated markers, as they are frequently present in low amounts and most of them have a molecular weight <30 kDa. To overcome this obstacle, the removal of high-abundance urinary proteins (e.g., uromodulin, albumin, and immunoglobulin G) and different depletion strategies (e.g., various sorbents for the removal of unwanted proteins) have been proposed in recent years to improve the visibility of rarer species, followed by subsequent protein identification by two-dimensional electrophoresis (2DE) and mass spectrometry (MS) analysis [15,16]. Since, to the best of our knowledge, this type of investigation has not yet been performed with urine from patients exposed to ESWL, with the aim of identifying changes in the protein pattern induced by lithotripsy and, thereby, revealing promising biomarkers useful in the early detection of ESWL-induced renal damage, in this study, we examined the composition of low-abundance urinary proteins from patients with nephrolithiasis before (baseline) and 24 h after exposure to ESWL, taking a proteomic approach. Protein profile analysis clearly demonstrated that the ESWL technique caused significant differences in the urinary protein composition of these patients.

## 2. Materials and Methods

### 2.1. Patients

Eighty patients with a proteinuria value less than 20 mg/mL were recruited for the present study. The validity of the sample number was verified using PASS 2005 software (Kaysville, UT, USA) with an alpha-type error of 5% and a beta-type error of 20%. We studied normotensive male patients (diastolic pressure less than 90 mm Hg), 41 ± 4 years old, with renal stone/s less of than 2 cm. All patients were hospitalized during the study. Patients with a serum creatinine value greater than 120 µmol or exposed to anesthesia and/or antioxidant therapy, as well as those with diabetic nephropathy, obesity (BMI higher than 30 kg/m^2^), coagulopathies, cancer, autoimmunity diseases, and uncompensated hypertension, were excluded from the study. The study protocol was approved by the Joint Ethic Committee of the G. D’Annunzio University of Chieti-Pescara-Local Health Authorithy 2 (ASL2) (No 9_10072014). Informed consent was obtained from all patients during enrollment. Before starting treatment, patients underwent standard assessments of hematological and biochemical blood parameters, as well as abdominal computed tomography (CT) without a contrast medium. Routine laboratory analyses were performed using established techniques.

### 2.2. ESWL Procedure

The ESWL procedure was performed as an outpatient procedure with the application of standard analgesia. All patients received a number of 2000 shock waves in a single session for an average energy of 350 kJ, at 60 strokes/minute, using the Sonolith 4000 plus extracorporeal lithotripter (EDAP Technomed Italia, Rome, Italy).

### 2.3. Sample Preparation

For each patient, 70–120 mL of urine was collected 2 h before and 24 h after ESWL treatment. After collection, each urine sample was adjusted to pH 7 with 1 M NaOH, then 1.5 mM NaN_3_ and a protease inhibitor cocktail (GE Healthcare, Munich, Germany) were added to prevent bacterial growth and inhibit endogenous protease activity, respectively. Subsequently, samples were centrifuged at 4 °C for 30 min at 3000× *g* to remove cellular debris, salts, and contaminants. Supernatants were collected and desalted with the Amicon device with a cut off of 3 kDa (Merck Life Science, Milan, Italy), concentrated up to ten-fold, and then stored at -80 °C until use.

To extract low-abundance proteins, an equalization protocol was applied to each sample using a commercially available enrichment method (ProteoMiner kit, Bio-Rad Laboratories, Segrate (MI), Italy) [17]. Proteins bound to the beads of the Proteominer kit were eluted using 9M urea followed by a chaotropic reagent mixture composed of 1.5M Trizma, 2M urea, 7M thiourea, and 4% 3-[(3-cholamidopropyl)dimethylammonium]-1-propanesulfonate (CHAPS, Sigma Italia, Opera, MI, Italy). Total protein concentration was estimated using a BCA protein assay (Pierce, ThermoFisher Scientific, Milan, Italy) according to the Bradford method [18].

### 2.4. Two-Dimensional Analysis

For the 2DE run, urine samples obtained from all 80 patients and enriched as described above were distributed into eight cohorts, each consisting of ten different samples, which were pooled to optimize the results. From each of the latter samples, 150 µg (for analytical gels) and 500 µg (for preparative gels) were used to perform the 2DE analysis according to the procedures described previously [19]. For each cohort, 3 independent runs were performed. A reference gel was created for each cohort by matching the 2D maps of the 3 individual electrophoretic runs while a master gel was then prepared by matching all reference gels for both pre- and post-ESWL treatment (see Supplementary Figure S1).

A comparative proteomic analysis was performed on the latter using Image Master 2D platinum software (6.0 version) to determine the quantitative changes in protein expression after ESWL treatment. Briefly, after background subtraction, the intensity volume of each spot was normalized to the total intensity volume. Additionally, synthetic gels were created using Image Master 2D platinum software that included all common spots in the 2D map of each cohort (see Figure 3). Changes in proteome expression resulting from the ESWL treatment of patients were more clearly revealed when comparing synthetic gels obtained from the urine of patients pre- and post-ESWL (as reported in Figure 4).

### 2.5. Protein Digestion and MALDI-TOF/TOF-MS Analysis

All protein spots chosen for their significantly different intensity levels between the studied groups were excised from 2D gels and analyzed by the peptide mass finger printing (PMF) approach with an MALDI-TOF/TOF spectrometer. Protein spots were picked from the gel, and each of them was first washed with 100% ethanol and 100 mM ammonium bicarbonate (NH_4_HCO_3_) and then incubated at 56 °C in 100 µL of 50 mM NH_4_HCO_3_ supplemented with 10 mM dithiothreitol (DTT, 60 min) and immediately after at room temperature in 100 µL of 50 mM NH_4_HCO_3_ plus iodoacetamide (30 min in the dark). Finally, the gel was incubated in 50 mM NH_4_HCO_3_ containing trypsin at 37 °C overnight [20]. This peptide extract was applied to a C18ZipTip (Millipore, Bedford, MA, USA), rinsed (0.1% trifluoroacetic acid, TFA), and eluted directly onto the MALDI target in 0.5 μL of a saturated solution composed of 1:1: α-cyano-4-hydroxycinnamic acid/0.1% TFA. MS analysis was performed using an Autoflex Speed mass spectrometer (Bruker Daltonics, Bremen, Germany), as previously described [21]. A mixture of reference peptide fragments (i.e., bradykinin fragment 1–7, 757.39 *m*/*z*; angiotensin II 1046.54 *m*/*z*; ACTH fragment 18–39, 2465.19 *m*/*z*; GluFibrinopeptide B 1571.57 *m*/*z*; and porcine renin tetradecapeptide substrate 1760.02 *m*/*z*) was used for external high-precision calibration (HPC), while trypsin autodigestion products (843,50 *m*/*z*, 1.046,56 *m*/*z*, 2.212,11 *m*/*z*, and 2.284,19 *m*/*z*) were used for internal mass calibration. Samples were also analyzed using LIFT MS/MS from the same target. The most abundant ions per sample were chosen for MS/MS analysis. Analyses were performed in positive LIFT reflectron mode [22].

### 2.6. Database MS/MS Searching

After MS acquisition, each spectrum obtained from PMF was searched in the NCBIn protein database by the Mascot search engine. Multiple parameters (e.g., PMF enzyme, trypsin; fixed modification, carbamidomethylation (Cys); variable modification, methionine oxidation; mass values, monoisotopic; ion charge state set to +1; maximum miscleavage set to 1; mass tolerance of 100 ppm for PMF and 0.6–0.8 Da for MS/MS) were used in this search to compare the experimentally determined tryptic peptide masses with the theoretical peptide masses calculated for proteins from the database. Additionally, after the automated assessment of the search results, the samples were automatically submitted for LIFT TOF/TOF acquisition for the validation of the PMF data analysis. A maximum of four precursor ions per sample were chosen for MS/MS analysis. An additional protein database search, using combined PMF and MS/MS datasets, was performed using SwissProt database (SwissProt_2012_03.fasta) via BioTools 3.2 (Bruker Daltonik GmbH, Billerica, MA, USA) connected to the Mascot search engine. The Mowse probability score (the observed match between the experimental dataset and each sequence database entry is a chance event, *p* < 0.05) was used as a criterion for correct identification. Scores are reported as −10 Log_10_(*p*), where *p* is the probability. The lowest probability corresponds to the highest score and is reported as the best match. Typically, this is a score of around 70 for PMF and 30–40 for MS/MS search.

### 2.7. Protein Validation by Western Blot Analysis

Urine samples obtained as described in paragraph 2.2 were used. Protein samples (usually 50 μg), after dilution in sodium dodecyl sulfate (SDS)-bromophenol blue buffer, were boiled (5 min), run on 12% SDS polyacrylamide gels, and subsequently transferred on polyvinylidene fluoride (PVF) membranes, where they were blocked by a mixture containing PBS, 0.1%, Tween20, and 5% nonfat milk (Bio-Rad Laboratories) for 2 h at 4 °C. Then, these membranes were incubated (overnight, 4 °C) with primary polyclonal antibodies (rabbit anti-matrilysin, dilution 1:1000; rabbit anti-alpha1-antitrypsin, dilution 1:500; and goat anti-clusterin, dilution 1:1000, all purchased from Abcam, Cambridge, UK), and subsequently (1 h, room temperature) with goat anti-rabbit or rabbit anti-goat HPR-conjugated secondary antibody (final dilution 1:5000, Bethyl Laboratories Inc.; Montgomery, TX, USA). Immunocomplexes were visualized by chemiluminescence (ECL) detection system (GE Healthcare Life Sciences, Milan, Italy) and quantified by densitometric analysis (ImageJ software, 1.54k 15 September 2024; U.S. National Institutes of Health, Bethesda, MD, USA).

### 2.8. Bioinformatic Analysis of Proteomic Data

Protein ontology classification was performed by importing proteins for analysis via the Gene Ontology (GO) classification system (http://www.expasy.org/). The identified proteins were analyzed and grouped based on their associated molecular functions and related pathways using the PANTHER database (https://www.pantherdb.org). Furthermore, using the STRING software (http://string-db.org/), chosen as the source for protein–protein interactions, the interconnections existing between most of them were highlighted by creating networks and forming clusters based on known direct and indirect interactions described in the literature. We also applied a pathway enrichment analysis to determine the different pathways of the proteins with statistical significance included in the networks identified by STRING analysis according to the KEGG (Kyoto Encyclopedia of Genes and Genomes) database. Data analysis was performed using the ShynyGO 0.82 workbench (http://bioinformatics.sdstate.edu, accessed on 15 April 2025).

### 2.9. Statistical Analysis

Reference gels and synthetic gels were used to assess the presence of, and difference in, protein levels. Background subtraction was performed, and the intensity volume of each spot was normalized by the total intensity volume (by adding the intensity volumes obtained from all spots within the same 2D gel). All quantitative data are reported as means ± SEM values. The intensity volumes of individual spots were matched between different gels and then compared between groups by multiple comparisons using one-way analysis of variance (ANOVA). A *p* < 0.001 was considered statistically significant. Significantly different protein spots were subjected to in-gel tryptic digestion and MS identification. Additionally, statistical comparisons between values from different treatments in the same model were calculated using Student’s *t*-test for unpaired data by GraphPad Prism software (version 6.0). *p*-values were corrected for multiple comparisons where appropriate.

## 3. Results

### 3.1. Urine Sample Collection and Removal of High-Abundance Proteins

Urine was obtained from enrolled patients 2 h before and 24 h after their exposure to ESWL. Before starting the proteomic analyses, collected samples were concentrated up to ten-fold, since protein concentration is usually low in normal urine, and then subjected to the Proteominer protein enrichment kit to optimize the samples, thus excluding the interference with those proteins that are normally present in abundance in urine [23]. The fractions eluted by Proteominer were distributed in eight different cohorts, each formed by pooling the urine of ten patients. This passage was crucial to obtain a higher protein concentration and to reduce the heterogeneity of urinary samples.

### 3.2. Urine Proteomic Analyses

As anticipated in the Methods section, representative urinary master gels were obtained for each examined condition, pre- and post-ESWL, by combining the 2D gel electrophoresis maps of each cohort (Figure S1). Representative 2D master gels resolved 945 ± 65 spots and 1106 ± 130 spots for pre- and post-ESWL, respectively, allocated in the pH range 4–7 and a molecular mass ranging from 10 to 180 kDa.

**Figure 1:**
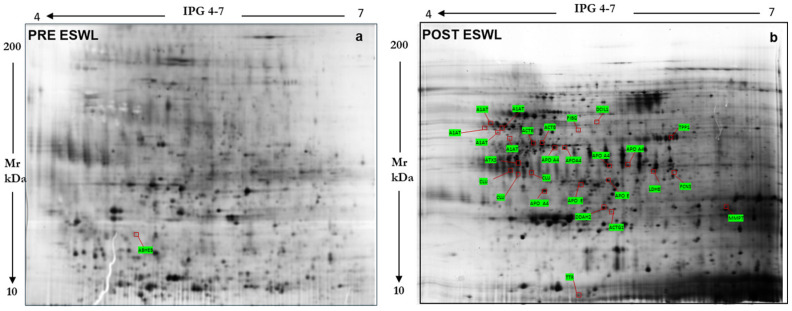
Reference map of the urinary proteome annotating 24 proteins identified by mass spectrometry, whose levels were increased in urine samples from patients 2 h before ((**a**) left panel) and 24 h after their exposure to ESWL ((**b**) right panel).

**Figure 2:**
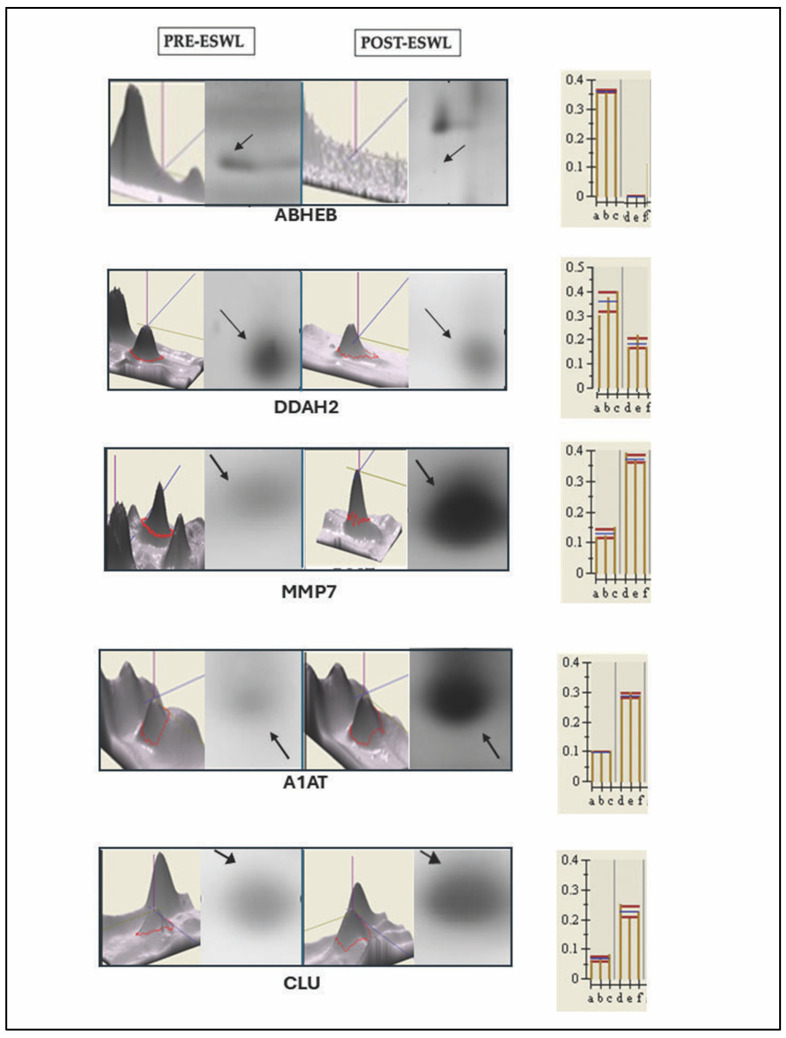
Examples of some proteins whose content was modified by the exposure of patients to ESWL (post-ESWL) compared to the pre-exposure condition (pre-ESWL), as identified by 2DE electrophoresis and subsequent MS analysis. Black arrows indicate the spots of the same protein in pre- and post-ESWL urine. On the left of each panel, the peaks of each protein measured in MS are reported, while, on the right, the magnification of the relative spot obtained from 2D gel and histograms with the corresponding intensity volume values (*p* < 0.0005) are given. ABHEB, putative protein-lysine deacylase ABHD14B Ab-hydrolase; DDAH2, N(G),N(G)-Dimethylargine dimethylaminohydrolase 2; MMP7, Matrix metalloproteinase 7, also known as matrilysin; CLU, clusterin; A1AT, alpha1-antitrypsin.

The comparison of these representative master gels and MS analysis (Figure 1 and Figure 2) allowed us to identify one protein exclusively present in pre-ESWL urine, namely, abhydrolase domain-containing 14B (ABHEB, also known as putative protein-lysine deacylase ABHD14B), while twenty-four proteins were detected in both pre- and post-ESWL urine samples, although they were differentially regulated, as reported in Table 1 (see below). Indeed, in post-ESWL samples, most of the identified proteins were upregulated, while six were downregulated (DDAH2, ACTG1, UBQL4, C3, DC1L1, and FIBG) compared to the protein levels found in pre-ESWL urine (for example only, see Figure 1b). Among the upregulated proteins, three were present as isoforms of the same protein, namely, A1AT, APOE, and APOA4.

**Figure 3:**
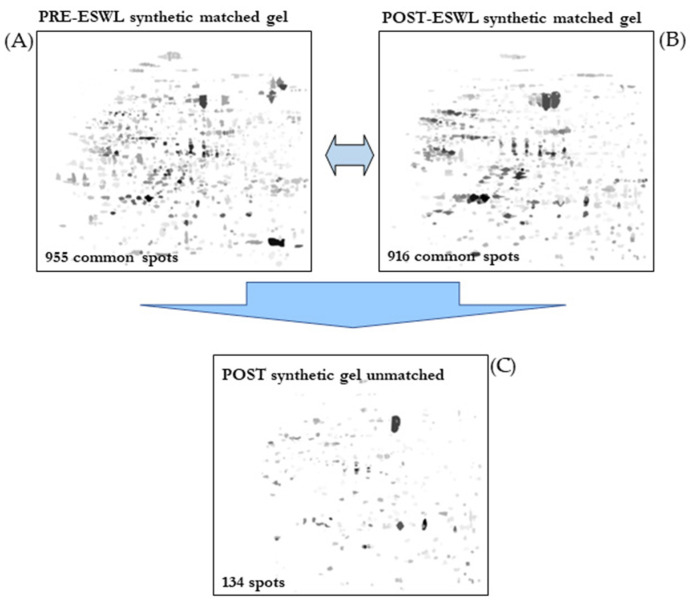
Comparative analysis of synthetic gels obtained for pre-ESWL (**A**) versus post-ESWL (**B**) conditions, which allowed the resolution of 134 unique spots in the post-ESWL condition (**C**).

Furthermore, since we noticed some intervariability between patient samples, to minimize this drawback and to further characterize the urinary proteome, we constructed two synthetic gels representing the spots common to the condition under examination, one for the PRE-ESWL condition and the other for the POST-ESWL condition (Figure 3A,B). The first showed 955 common spots while the second showed 916 common spots. Furthermore, the comparison of the matched post-ESWL synthetic gel with the 2D map of each pretreated cohort reveals 134 spots (Figure 3C), 50 of which can be unambiguously assigned to unique proteins that can be considered secondary to the lithotripsy treatment (Table 2).

Again, many of these proteins were isoforms, cleaved polymers, or precursors of the same protein as in the case of alpha–1-antitrypsin (A1AT), apoliprotein A I (APOA1), apolipoprotein IV (APOA4), apolipoprotein E (APOE), alpha enolase (ENOA), complement protein 9 (C9), complement factor B (CB), and glutamyl-peptide cyclotransferase (QPCT). However, the isoforms of A1AT, APOE, and APOA4 and the sequence of other proteins such as transthyretin (TTR) or complement 3 (C3) reported in Table 2 are different from those previously identified in the reference gels obtained from the different patient cohorts and reported in Table 1, while the sequence of ACTB and that of canonical proteins such as A1AT (P01009), APOA4 (P06727), and APOE (P02649) are the same in both tables.

**Figure 4:**
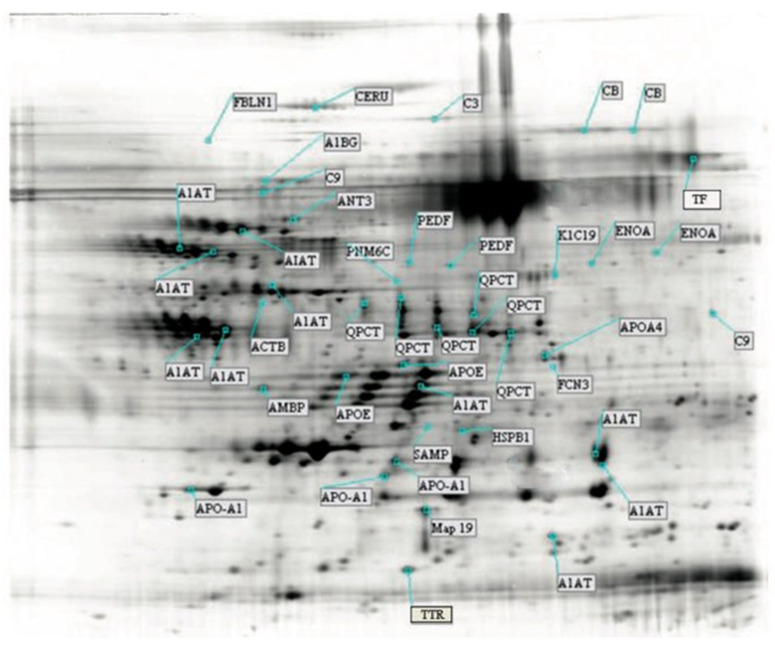
Post-ESWL synthetic gel urinary proteome map annotating 50 unique proteins identified by mass spectrometry. Protein names with their abbreviations are those reported in Table 2.

In summary, through the procedures and analyses described above, we resolved a total of 75 (25 plus 50) proteins from the urine of patients with nephrolithiasis. Of these, 50 were found only in urine from patients after their exposure to ESWL, while 24 were present in urine samples obtained from patients after exposure to ESWL and were significantly up- or downregulated in post-ESWL urine compared to pre-ESWL urine (Figure 4). However, as noted above, many of the identified proteins were isoforms of the same protein.

### 3.3. Validation of the Protein Sequence and Urinary Content, Respectively, by LIFT-MALDI TOF (MS/MS) and Western Blot Analyses of Some Peculiar Proteins Identified by MS and Selected Among the Others in Post-ESWL Urine Samples

Using LIFT technology (as described in the Methods section), we analyzed a number of proteins identified by MS and reported in Table 1 and Table 2. With this method, we obtained parental ion masses from PMF spectra and the high Tof-Tof Score values unequivocally confirmed the unique peptide sequences (reported in red) for each protein (Table 3). For example, here, we report the analysis of three proteins that were shown to be highly upregulated in post-ESWL urine samples.

**Figure 5:**
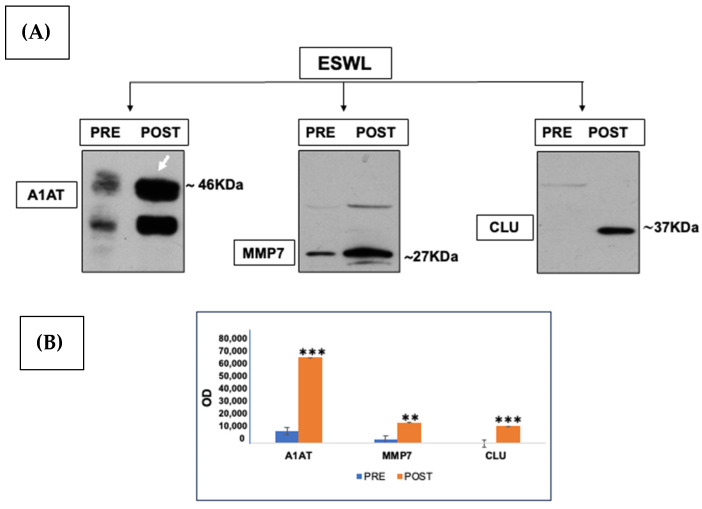
(**A**) Representative Western blots showing changes in alpha-1-antitrypsin (A1AT) (where the white arrow indicates the appropriate immune band while the lower one is likely an isoform of the same protein), clusterin (CLU), and matrix metalloproteinase 7 (MMP7) content induced by ESWL treatment in patients with nephrolithiasis as previously identified by MS and reported in Table 1 together with other proteins. Protein lysates (30 µg) were loaded for each electrophoretic lane. The original blots are reported in Figure S2 in the Supplemental Material. (**B**) Bands in the immunoblots were quantified by densitometric analysis, performed as reported in the Methods section, and their values are reported as optical density (OD) on the y axis of the histograms. ** *p* < 0.01, *** *p* < 0.001: statistical significance vs. post-ESWL samples (Student’s t test). N. of examined urine samples = 3 for each condition (pre- and post-ESWL), each of which is a pooled sample of urine specimens collected from 10 patients before and after their exposure to ESWL.

To further validate the results of the proteomic analysis in relation to the changes in the expression levels of urinary proteins after ESWL treatment in patients, Western blot analysis was performed on the selected proteins reported above. This technique also confirmed the identification of these proteins obtained by MS (Figure 5A,B). Therefore, with these two methods, we are confident that the indicated proteins have an intact amino acid sequence and could be used as putative biomarkers of ESWL-induced changes.

### 3.4. Computer Analysis of the Biological Functions and Related Pathways, as Well as Possible Networks, Between the Urinary Proteins Whose Levels Were Modified by Exposure of Patients to Lithotripsy

**Figure 6:**
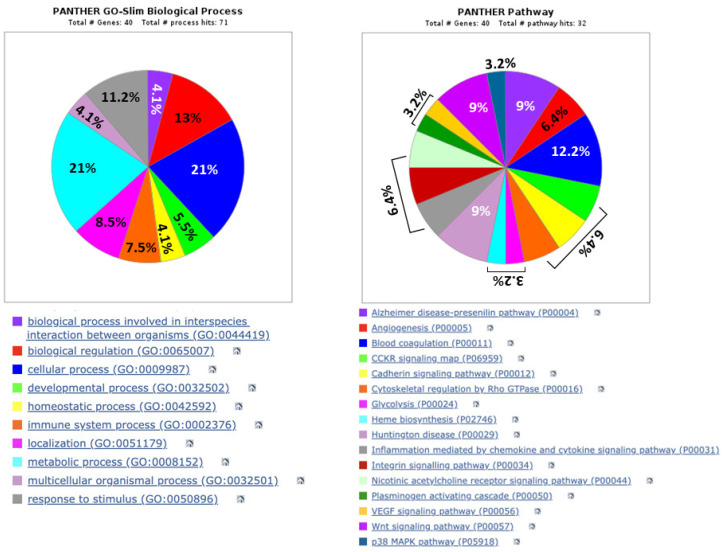
Biological processes and pathways of differentially expressed proteins in post-ESWL urine samples, as revealed by MS analysis, were obtained using the PANTHER database. The percentage of proteins grouped according to the biological processes and pathways in which they are mainly involved, which are identifiable by the colored squares below the pies, are reported inside the pies in the left panel and only some in the right panel, where other percentages are reported above the square brackets, meaning that each colored triangle has the same percentage.

The composition of the assigned proteome was characterized based on biological functions and related molecular pathways, annotated in the Gene Ontology database (GO: wwe.expasy.org _UNIPROT) (Figure 6).

With this analysis, it was possible to highlight the biological processes of dysregulated proteins in urine samples from the exposure of patients to ESWL. Most of them usually play a role in metabolic (21%) or cellular (21%) processes, the regulation of biological functions (13%), responses to stimuli (about 11%), and immune system processes (7.5%). The activities of all these proteins are implicated in a variety of pathways that modulate inflammation and related signals (chemokines, cytokines, and Wnt and p38 MAPK pathways), angiogenesis and the VEGF pathway, blood coagulation, cell cytoskeletal modeling, and cell–cell interactions. Unexpectedly, some of them are related to pathways implicated in neurodegenerative disorders such as Alzheimer’s or Huntington’s diseases, as emphasized in the Discussion Section. Finally, a large portion of the assigned proteins appear to be cytoplasmic, some from the endoplasmic reticulum, but there are no nuclear proteins, mainly due to the absence of intact cells in the urine.

**Figure 7:**
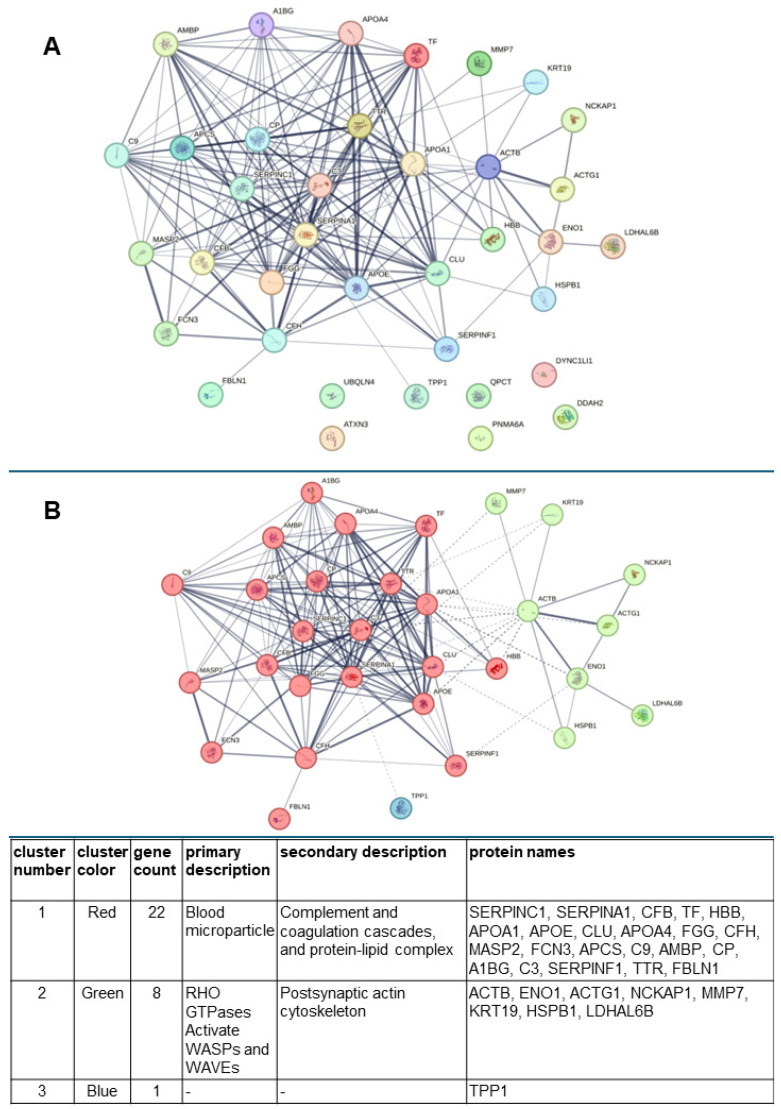
Computer analysis performed using the STRING database to evaluate the mutual interactions between the proteins identified by MS analysis and reported in Table 1 and Table 2. (**A**) This analysis highlighted a close interaction between the proteins identified by MS in the urine of patients after ESWL and dysregulated by this treatment. The colors of the circles in this panel were randomly selected by the STRING database. (**B**) STRING analysis divided these proteins into three different clusters, where the genes corresponding to the identified proteins are reported inside circles labeled with different colors and whose composition is reported in the Table below the graph. The name of the proteins encoded by the genes is reported in Table S1, in the Supplementary Material.

Using the STRING database (http://string-db.org, accessed on 25 February 2025), the interconnections existing between all proteins, identified by MS, that were dysregulated in urine due to patient exposure to ESWL were constructed (Figure 7A).

Interestingly, this analysis revealed a *p*-value for protein–protein interaction (PPI) enrichment <10^−16^, meaning that the proteins have more interactions with each other than would be expected from a random set of proteins of the same size and degree distribution extracted from the genome. Such enrichment indicates that the proteins are at least partially biologically connected as a group. Furthermore, by continuing the analysis in the STRING database, we highlighted the formation of three main clusters recognizable by the different colors of the spheres (red, green, and blue) in which the genes encoding the selected proteins (reported in Table S1 of the Supplemental Material) are indicated (Figure 7B). These represent the nodes, which are associated with each other by edges to indicate their interaction, while thicker lines represent stronger associations. The main clusters with the related color are reported in the table above the figure, as derived from the STRING analysis. Cluster n. 1 is the largest, comprising most of the identified proteins in the post-ESWL urine, which can be found in blood microparticles, participating in the complement and coagulation pathways, or in the protein–lipid complex. In contrast, cluster n. 2 contains structural proteins of cytoskeleton. STRING analysis revealed a significant enrichment in the RHO GTPases activating the WASP and WAVE pathway, which is known to play a key role in cell migration and invasion, while cluster n. 3 includes only one protein, tripeptidyl-peptidase-1 (TTP1), a serine protease. Most of these aspects are analyzed in more detail in the Discussion Section.

**Figure 8:**
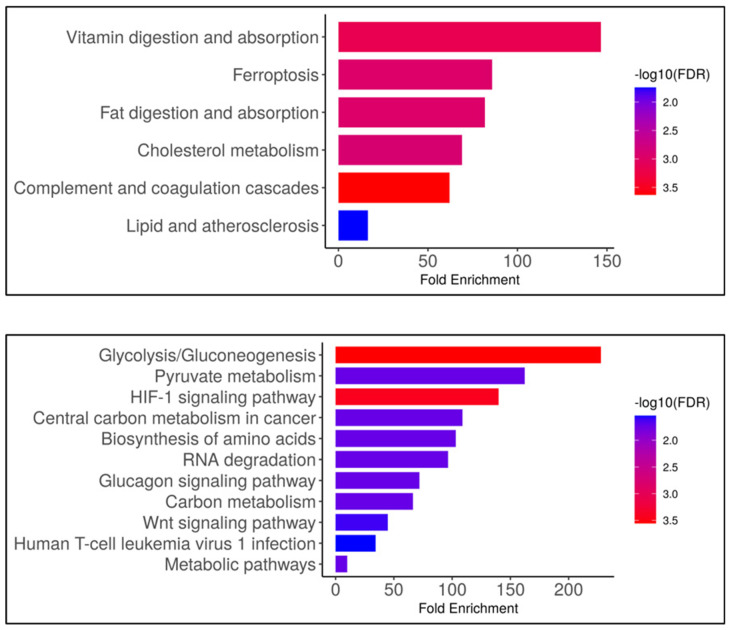
KEGG pathway enrichment analysis of proteins in clusters n.1 (upper histograms) and n. 2 (lower histograms). The abscissa represents the fold of enrichment of the genes under each pathway classification while the ordinate represents the enrichment pathway. On the right in each panel, the colored bar ranging from blue to red represents the enrichment expressed as –log10 from decrease to increase in proteins in the groups.

Finally, an additional pathway enrichment analysis of the proteins forming the two main clusters was performed using the KEGG database (Figure 8).

This analysis highlights that the proteins included in cluster n. 1 are involved not only in complement-and-coagulation-related or lipid-turnover-related pathways but also in vitamin metabolism and ferroptosis, a cell death pathway involving iron accumulation and lipid peroxidation. The same analysis showed that the proteins of cluster n. 2 are also implicated in cellular metabolism, mainly in carbohydrate and protein turnover, or in pathways such as HIF-1 signaling, related to the regulation of the cellular response to oxygen levels, or the Wnt signaling pathway, involved in the control of cell fate and tissue homeostasis. However, the same protein, MMP7, which regulates Wnt signaling, could also be involved in human T-cell leukemia virus type 1 infection.

## 4. Discussion

The aim of this investigation was to analyze urinary protein composition in order to determine changes in the protein profile caused by ESWL and, possibly, putative biomarkers of renal damage. To achieve this goal, the first step was to enroll an adequate number of patients with nephrolithiasis to be treated with ESWL by performing a careful selection based on their health condition. They were all male, since in a preliminary series of experiments, female urine showed higher variability. Furthermore, it should be noted that 2DE separated a relatively low number of spots from urine samples obtained from those patients, and MS identification of proteins occurred with poor resolution, likely due to the presence of high levels of proteins, most of which were of plasma origin. Therefore, the whole analytical phase was preceded by a methodological setup phase during which all urine samples, collected from subjects with nephrolithiasis treated with ESWL in the pre-treatment (2 h) and post-treatment (24 h) conditions, were subjected to the Proteominer enrichment procedure to deplete them of highly abundant proteins. With this technique, we obtained a good resolution of the less abundant proteins by electrophoretic runs, which should better reflect the changes in urinary protein pattern caused by patients’ exposure to ESWL. Indeed, when we adopted this technique for sample preparation, it proved to be an optimal method with a high degree of reproducibility and efficiency in terms of overall protein yield [24]. Furthermore, since there was a large heterogeneity between the two-dimensional maps obtained for each sample and condition, it was necessary to create two pools of samples, consisting of control urine samples (pre-ESWL) and post-ESWL treatment urine samples, respectively, for which the image analysis and identification of each protein change were repeated, referring to synthetic gels built on specific proteomic data of the two conditions under examination and, above all, free of interindividual variables. This procedure allowed us to determine, with a certain statistical rigor, a greater number of changes in the protein level in the urine in the post-ESWL condition. Overall, with the above strategy, we were able to demonstrate that lithotripsy significantly modified the urinary protein profile with regard to the low-abundance protein composition, corroborating, at the biochemical level, the notion that significant morphological and functional changes can occur in the kidney with ESWL treatment [4,14,25,26,27,28,29].

First, it is noteworthy that only one protein, namely, putative ab-hydrolase domain-containing protein 14 (ABHEB), was present in pre-ESWL samples and no longer detectable in patient samples after ESWL. Since this enzyme is a papillary signaling protein associated with immunity and defense, which has been found to be significantly upregulated during osmotic stress [30], our finding suggests that the renal osmotic stress defense capacity is strongly reduced after lithotripsy.

In contrast, our MS analysis identified a large number of de novo expressed or upregulated proteins in post-ESWL samples compared to the levels detected in pre-ESWL urine. STRING analysis applied to these proteins showed a close relationship among them, allowing the formation of three main clusters.

Cluster n. 1 is the richest, comprising 22 proteins of those reported in Table 1 and Table 2. One of the most interesting is A1AT, a protease that protects tissues from enzymes released from inflammatory cells, which was identified in our experiments with a large number of isoforms that are post-translation modifications of the same protein encoded by the SERPINA1 gene. The identification of A1AT was not surprising since many previous urinary proteomic studies have indicated this protein as one of the major biomarkers for the diagnostic and prognostic evaluation of different renal diseases [31]. Note that A1AT occupies a central position in cluster n.1 (Figure 5), where it is linked to several other proteins dysregulated by ESWL treatment. Among them, clusterin (CLU) is usually related to lipid utilization, cell aggregation, and adhesion. Its increased expression has been found in several types of nephropathy [32] and has been identified as one of the possible novel biomarkers of “subclinical AKI” [33]. In cluster n.1, A1AT and CLU are also interconnected with complement-related and protein–lipid-complex-related proteins. One of these is APOE. It can activate several proteins in the classic or alternative pathways of the complement cascade, which participate in the innate immunity required for host defense but are also mediators of inflammation and, therefore, various forms of disease and injury [34]. Accordingly, the presence of increased levels of different complement factors (H, C3, C9, CB) in post-ESWL urine, possibly activated by APOE, is not surprising, confirming that the complement cascade is involved in renal injury [35]. C3, in particular, is a key molecule of this system that can be produced locally and activated in the kidney, where its increased levels are usually seen as being related to nephropathies; however, even lower levels suggest intra-renal inflammation and have been related to poor renal outcomes [36,37]. Since we found, in post-ESWL urine samples, several C3 isoforms, which were both down- and upregulated, it is reasonable to suspect that they may all contribute to renal damage. Furthermore, C3 and other complement factors can also be activated by leptin pathways, which in turn are stimulated by MAP19 (human MBL-associated protein 19), the short isoform and the alternative splice variant of MBL-associated serine protease 2 (MASP-2) that we found induced de novo by the ESWL procedure. Notably, in urine and uEVs, MAp19 is the dominant form and the renal tubule damaged due to ischemia represents an example of local complement activation following the presence of damaged renal cells [38,39]. Interconnected with the complement system in cluster n.1 is antithrombin III (ANT3 codified by the SERPINC1 gene), which, in addition to its known anticoagulant activity, exhibits anti-inflammatory properties, showing a slight increase in urinary levels in the acute phase of glomerulonephritis, but a large increase during the relapse phase of nephrotic syndrome, as reviewed in [40]. 

Additional apolipoproteins in the same cluster include apolipoprotein A-I (APOA1) and apolipoprotein A-IV (APOA4). APOA1 is the major protein component of HDL particles, which are crucial in reverse cholesterol transport, while APOA4 may play a role in the secretion and catabolism of chylomicrons and VLDL. Both exhibit antioxidant and anti-inflammatory activity, preventing thrombotic phenomena and protecting organs such as the heart, brain, and kidneys. However, proteomic analysis has previously found that increased levels of APOA1 may represent a promising biomarker for the early diagnosis of AKI after percutaneous coronary intervention in elderly patients [41]. In contrast, APOA4 concentrations are markedly increased in chronic kidney disease (CKD), mainly in dialysis patients, resulting in a potential early marker of renal impairment that can predict CKD progression [42]. 

Other well-interconnected proteins in cluster n. 1 are ceruplasmin (CERU, encoded by the CP gene) and serum amyloid P-component (SAMP encoded by the APCS gene). CERU has previously been identified by MS analysis among the upregulated glycoproteins in the urine of CDK patients, together with antitrombin III, glutamyl-peptide cyclotrasferase (QPCT), and tripeptidyl peptidase-I (TPP1) [43], as we also detected in post-ESWL urine samples, although these proteins are not included in the same cluster of CERU. These results are intriguing since TPP1, which forms cluster n. 3 of our network, and the ataxin 3 variant (ATX3) and the various QPCT isoforms, are mainly involved in the pathophysiology of debilitating neurodegenerative conditions or cancer; for example, see [44,45,46,47,48]. Doubts are also raised by the increased presence of the paraneoplastic antigenic protein 6C (now recognized as PNMA6A), included in the paraneoplastic Ma family, since no function or involvement in renal pathologies has been identified for this protein and its physiological function is still unclear [49]. On the contrary, SAMP/APCS belonging to the pentatraxin family is able to regulate many aspects of the innate immune system and has been recognized as the most predictive marker of experimentally induced or lupus nephritis [50].

Finally, some upregulated/de novo induced proteins in urine samples from ESWL-treated patients are located in the external part of cluster n.1, although they are connected to the main proteins mentioned above. Among these is alpha-1B glycoprotein (A1BG), a recognized marker for autoimmunity and cancer, whose induction in post-ESWL samples may be due to early and specific alterations in urinary glycoprotein excretion, as found in diabetic nephropathy [43]. As for alpha 1-microglobulin (AMBP), a low-molecular-weight heme-binding antioxidant plasma protein, once readily filtered through the glomerulus, it is reabsorbed and catabolized by the proximal tubular cells [51,52]. Accordingly, its increased presence in post-ESWL urine could indicate proximal tubular dysfunction and thus impaired tubular functions. Furthermore, AMBP, together with pigment epithelium-derived factor (PEDF, also known as SERPINF1) and hemoglobin subunit beta (HBB), has also been found in the plasma of patients with reduced glomerular filtration due to CKD [53]. In turn, many hemoglobin fragments have been found to be increased in patients with vasculitis or IgA nepfropathy (IgAN), which likely reflects an important glomerular inflammation [54]. As for the other proteins, serotransferrin (TF) has been correlated in rat and human urine samples with a risk of renal damage induced by subclinical tubular alterations [55], while Fibulin 1 (FBLN1) could be another possible biomarker of kidney injury, as demonstrated by proteomic studies performed both on sera of patients with different renal diseases [56] and on urinary extracellular vesicles isolated from the urine of patients with medullary sponge kidney disease [57]. Furthermore, Ficolin-3 (FCN3) is an important member of the fibrinogen family and an activator of the complement lectin pathway that, by causing pro-inflammatory effects, contributes to the onset/progression of inflammatory diseases, including nephritis. Very recently, elevated levels of FCN3, together with galectin-3, have also been found in sera of patients with AKI, following complicated sepsis and correlated with the severity of renal damage [58]. Finally, increased levels of transthyretin (TTR), also known as prealbumin, are relevant since elevated serum levels of TTR have been found in CKD, and, in the presence of tubular damage, there may be a leakage of TTR in the urine [59,60]. Therefore, all these proteins may also act in an acute-phase/immune-stress response in CDK subjects and are probably able to play a similar role after patient lithotripsy.

Further data that deserve to be discussed concern the upregulation of the proteins grouped in a second smaller cluster of seven proteins labeled by green circles that is, however, interconnected with cluster n.1. Among these is cytoplasmic actin 1 (ACTB), although the increased expression levels of this protein are counterbalanced by the observed downregulation of cytoplasmic actin 2, also known as ACTG1. Both proteins are components of the cytoskeleton but are localized on different chromosomes, and their protein chain differs by four residues at the N-terminus. These features likely account for differences in their location into actin structures within the same cell, as well as in their functions, with ACTB filaments being mainly involved in cell contraction processes while ACTG1 (*or* γ-actin) is enriched in stress fibers and plays a key role in endothelial cell motility, chemotaxis, and angiogenesis [61]. Notably, the Wiskott–Aldrich syndrome (WASP)/WAVE family of proteins also controls actin arrangement and function in the kidney, where it plays a key role in Na^+^ reabsorption by the aldosterone-sensitive distal nephron [62]. Consequently, when the dysregulation of actin cytoskeleton occurs (as may be the case in our study) in renal epithelial cells or in renal podocytes, which are fundamental in preventing the filtration of large proteins/micromolecules and whose function is largely dependent on actin cytoskeleton, this can lead to ionic imbalance and/or foot process retraction and proteinuria [63,64]. Other proteins of the same cluster electrophoretically resolved from post-ESWL urine samples belong to acute-phase response proteins and/or mediators of the fibrotic reaction, as previously found, reinforcing the idea that ESWL may induce renal inflammation and acute trauma. They include δ-aminolevulinic acid dehydratase (δ-ALA-D or Heme-2) [65], heat shock protein beta-1 (HSPB1) [66], and enolase A (ENOA), with the latter implicated in calcium oxalate stone formation [67]. Additionally, keratin C 19 (KTC19), a member of the keratin family that was induced de novo in urine by the exposure of patients to ESWL, is a common finding in progressive renal diseases, even if early after disease onset [68]. Notably, this cluster incudes the fibrogenic matrix metalloproteinase-7 (MMP7), also known as matrilysin, which belongs to a superfamily of Zn-dependent metalloproteinases responsible for maintaining cellular matrix homeostasis [69]. It has been found to be elevated in renal allograft inflammation/injury, also acquiring moderate discriminatory significance for subclinical rejection [70]. Thus, its upregulation in our post-ESWL samples supports the idea that this protein may be indicative of renal tubular epithelial cells damage and that these tubular cell types, under the action of MMP7, are undergoing epithelial–mesenchymal transition [71,72]. Of note, the presence of lactate dehydrogenase A like 6B (LDHAL6B) found in post-ESWL urine also strengthens the belief that this binding protein may be related to human glomerulus damage, as found in the urine from mouse diclorofenac-injured kidney glomerulus [73] or in models of AKI and CKD [74]. In summary, all these data support the hypothesis that lithotripsy may damage renal function, although often only temporarily.

Before concluding, it should be emphasized that exposure of patients to ESWL also caused a significant downregulation of six proteins, which are mostly ubiquitous and important for normal renal function. Indeed, in addition to the ACTG1 and C3 proteins already mentioned, there are reduced levels of dimethyl arginine dimethyl amino hydrolase 2 (DDAH 2), which is usually present in large amounts in several intra-renal sites, including the glomerular vasculature [75]. This enzyme degrades asymmetric dimethyl arginine (ADMA), an inhibitor of nitric oxide (NO) synthase [76]. As a consequence of the decreased level of DDAH2 produced by ESWL exposure, ADMA may increase and in turn predispose patients to the progression of vascular disease and renal damage [77]. Less clear is the significance of another protein downregulated in post-ESWL urine, namely, ubiquilin-like protein or ubiquilin-4 (UBQL4). It belongs to the ubiquilin family, consisting of four members, and regulates protein degradation, mediating the transport of misfolded, mislocalized, or accumulated proteins to the proteasome and is also a key regulator of DNA. So far, members of this family and, in particular, UBQL4 have been associated with the most common cancers [78], while another member, namely, UBQLN1, has been shown to be critical in combating neurological disorders caused by protein aggregation, such as amyotrophic lateral sclerosis (ALS), Alzheimer’s disease, and Huntington’s disease, while its loss has so far been found in lung cancer patients and human cancer cell lines [79]. Finally, two other downregulated proteins were cytoplasmic dynein (DC1L1) and fibrinogen gamma (FIBG). DC1L1 is a key member of the dynein complex containing, in addition to light intermediate and light chains, dinein heavy chains that exhibit ATPase activity responsible for the cargo transport necessary for cellular function. It is also found in the intracellular vesicles of renal collecting ducts, where it colocalizes with aquaporin 2 (AQP2) water channels, which are critical for the control of renal water permeability via vasopressin. Therefore, the downregulation of DC1L1 could alter the filtering function of the kidneys [80]. On the other hand, FGG is a primary component of fibrinogen, which comprises several chains including two α-chains, two β-chains, and two γ-chains (FGG) interconnected with disulfide bonds. In addition to its important role in blood coagulation, fibrinolysis, and cellular and matrix interactions, fibrinogen is also involved in the inflammatory response and wound healing. Soresensen et al. used a unilateral ureteral obstruction model in fibrinogen knockout mice and found that compared to fibrinogen deficient homozygous mice (Fib−/−), heterozygous mice (Fib+/−) showed more severe interstitial fibrosis damage, with massive fibrinogen deposition in the renal interstitium [81]. However, the results are contradictory, as fibrinogen deficiency has been associated with higher mortality in renal ischemia/reperfusion injury in rats [82]. Thus, the role of fibrinogen in AKI remains controversial.

## 5. Conclusions

In conclusion, this is the first study to report the proteomic profile of low-abundance proteins in urine from patients with nephrolithiasis treated with ESWL. The results show that inflammatory, fibrotic, and antioxidative proteins are the major constituents confirming, at the biochemical level, that ESWL can produce inflammation and fibrotic processes. Indeed, most of those identified in post-ESWL urine samples have been previously proposed as possible biomarkers for renal tissue damage in both AKI and CKD. Therefore, most of them could also play a putative role as biomarkers of renal tubular and glomerular damage induced by patients’ exposure to lithotripsy, such as matrix metalloproteinase 7, alpha1-antitrypsin, and clusterin, which were remarkably increased in post-ESWL urine samples, as well as dimethyl arginine dimethyl amino hydrolase 2 and abhydrolase domain-containing protein 14, which were downregulated in the same urine samples. We are aware that further studies with a larger series and repeated measurements should be performed to clarify whether these proteins can be used to demonstrate renal damage and as urine biomarkers for the diagnosis and treatment of renal injury after ESWL. Nevertheless, we believe that the use of urine represents a non-invasive method that allows the early identification (24 h) of urine proteins that could serve as potential biomarkers to predict the severity or otherwise of the damage caused by lithotripsy and accelerate the application of the most appropriate clinical investigations and treatments.

## Figures and Tables

**Table 1:** Urinary proteins significantly changed after ESWL treatment as identified by spots on reference gels.

Protein Name	Abbreviation	Swiss- Prot/NCBInr AC ^a^	SCORE ^b^ _SC ^c^	Matching Peptides #	Theoretical Mr_pI	n-Fold Variation ^d^ (*p* < 0.001)
Alpha-1 antitrypsin variant	A1AT	P01009	61_19	7	46,751-5.43	+3.42
Actin, cytoplasmic 1	ACTB	gi|501885	155_46	20	42,058_5.29	+2.50
Apolipoprotein A-IV precursor	APOA4	gi|178757	126_35	18	45,353_5.33	+1.32
Ficolin-3	FCN3	O00602	47_18	5	33,404_6.20	+2.13
Apolipoprotein E	APOE	P02649	54_30	9	36,248_5.65	+1.69
Serpin peptidase inhibitor	A1AT	gi|15080499	62_23	8	46,864_5.36	+0.91
N(G),N(G)-dimethylarginine dimethylaminohydrolase 2	DDAH2	O95865	80_22	7	29,915_5.66	−2.40
Actin, cytoplasmic 1 G	ACTG1	gi|40226101	67_7	21	29,878_5.5	−2.12
Matrix metalloproteinase 7	MMP7	gi|348021	82_26	9	29,810_6.56	+4.18
Delta-aminolevulinic acid dehydratase	Hem2	P13716	40_19	6	36,728_6.32	+2.76
Ubiquilin-4	UBQL4	Q9NRR5	67_24	9	53,205_5.67	−1.88
Complement C3	C3	gi|78101268	88_15	8	187,148_6.02	−2.02
Hemoglobin subunit beta	HBB	P68871	59_40	5	16,102_6.75	+3.01
Alpha-1 antitrypsin	A1AT	gi|50363217	112_37	14	46,790_5.42	+1.66
Ataxin 3 variant	ATX3	gi|28951768	54_26	6	38,978_4.81	+1.27
Clusterin	CLU	gi|338305	110_41	12	37,002_5.74	+2.69
Apolipoprotein E	APOE	gi|178853	61_29	9	36,243_5.81	+1.78
Cytoplasmic dynein	DC1L1	Q9Y6G9	55_13	5	56,834_6.01	−1.93
Fibrinogen gamma	FIBG	gi|70906437	75_41	8	50,103_5.70	−1.18
Apolipoprotein A-IV	APOA4	P06727	66_12	23	45,371_5.28	+1.39
Lactate Dehydrogenase A like 6B	LDHAL6B	Q9BYZ2	131_36	14	41,943_5.86	+3.01
Tripeptidyl Peptidase-I	TPP1	gi|215261288	51_11	5	62,797_6.26	+1.19
Transthyretin	TTR	gi|212374952	66_56	5	13,799_5.35	+1.65
Apolipoprotein A4	APOA4	gi|93163358	123_37	17	45,371_5.28	+2.77

(a) AC represents accession numbers from the Swiss-Prot database. (b) Score is −10*Log(*p*), where *p* is the probability that the observed match is a random event; it is based on the Swiss-Prot database using the MASCOT searching program as MALDI-TOF MS data. (c) SC, sequence coverage. (d) n-fold variation indicates the changes in mean, normalized spot abundance value between pre- and post-ESWL conditions. The mean abundance value and its SEM were calculated from the normalized spot intensities from n = 8 replicate gels for each pre- and post-ESWL condition.

**Table 2:** Additional urinary proteins showing increased content in urine samples after ESWL treatment in patients, as revealed by MS analysis of peptides in post-ESWL synthetic gels compared to pre-ESWL gels.

Protein Name	Abbreviation	Swiss- Prot/NCBInr AC ^a^	SCORE ^b^ _SC ^c^	Matching Peptides #	Theoretical Mr_pI
Actin, cytoplasmic 1	ACTB	P60709	109_37	11	42,052/5.28
Alpha-1-antitrypsin	A1AT	P01009	190_5555	23	46,878/5.37
S Variant of Human Alpha1-Antitrypsin	A1AT	gi|231240	200_57	22	39,099/5.27
Alpha1-Antitrypsin	A1AT	gi|253723069	115_67	27	44,273/5.43
Cleaved Antitrypsin Polymer	A1AT	gi|7546268	200_55	19	37,622/5.43
Antitrypsin alpha-1 mutant	A1AT	gi|224224	229_45	22	46,873/5.35
Serpin-Proteinase Complex	A1AT	gi|83754916	132_36	14	40,041/5.20
Cleaved Antitrypsin P10 Pro	A1AT	gi|301598706	201_53	19	38,558/5.11
Cleaved Antitrypsin Polymer	A1AT	gi|7245932	98_54	17	36,612/5.44
A1AT Alpha-1-antitrypsin	A1AT	gi|28637	54_29	8	22,871/6.11
Cleaved Antitrypsin P10 Pro, P9-P6 Asp	A1AT	gi|301598708	76_27	9	38,474/5.16
Inhibitor BrCN fragment lI, alpha1 protein	A1AT	gi|223039	104_63	7	11,785/5.76
Alpha-1B-glycoprotein	A1BG	P04217	90_23	10	54,790/5.56
Alpha- 1- microglobulin	AMBP	P02760	59_23	7	38,999/5.95
Antithrombin-III	ANT3	P01008	226_46	25	53,025/6.32
Apolipoprotein A-I	APOA1	P02647	280_73	30	23,00/5.22
Chain A Human Apolipoprotein A-I	APOA1	gi|2914175	219_88	25	23,389/5.55
Truncated Human Apolipoprotein A-I	APOA1	gi|347447518	271_84	25	21,611/5.02
Apolipoprotein A-IV	APOA4	P06727	254_56	28	45,371/5.28
Apolipoprotein A-IV	APOA4	gi|563320	291_33	62	28,141/5.39
Apolipoprotein E	APOE	P02649	53_28	10	36,246/5.46
Apolipoprotein E precursor	APOE	gi|4557325	195_62	24	36,246/5.56
Alpha-enolase OS	ENOA	P03733	53_16	7	47,481/7.01
Crystal Structure of Human Enolase 1	ENOA	gi|203282367	89_23	10	47,350/6.99
Ceruloplasmin	CERU	P00450	245_28	28	122,983/5.44
Complement protein 9	C9	gi|179726	175_24	18	64,399/5.49
Complement component C9	CO9	P02748	98_13	10	64,615/5.43
Complement factor B	CB	gi|57209925	55_14	10	86,648/6.44
Complement factor B preproprotein	GB	gi|67782358	55_11	9	86,847/6.67
Human Complement Component C3	C3	gi|78101268	153_22	22	11,4238/5.55
Complement factor H	CFAH	P08603	267_25	35	14,3680/6.21
FicoIin-3	FCN3	O75636	82_36	11	33,395/6.20
Fibulin-1	FBLN1	P23142	56_16	10	81,268/5.07
Glutaminyl-peptide cyclotransferase	QPGT	Q16769	191_44	18	40,965/6.12
Glutaminyl Cyclase Mutant E201	QPCT	gi|185177689	195_54	19	37,590/5.78
Glutaminyl-peptide cyclotransferase	QPCT	gi|75766183	150_48	18	37,606/5.69
Glycosytated Human Glutaminyl Cyclase	QPCT	gi|345101018	127_40	15	37,916/5.96
Glutaminyl-peptide cyclotransferase	QPCT	Q16769	171_45	15	40,965/5.69
Glutaminyl CycIase Mutant	QPCT	gi|185177695	175_48	17	37,619/5.78
Glutaminyl CycIase with Glutamine	QPCT	gi|75766189	162_53	19	37,605/5.78
Heat shock protein beta-1	HSPB1	P04792	150_11	50	22,826/5.98
Keratin C 19	K1C19	P08727	61_19	8	44,079/5.04
Human MbI-Associated Protein 19	Map19	gi|50513645	103_44	8	19,531/5.44
Paraneoplastic antigen-like protein 6C	PNM6C	P0CW26	46_10	5	44,304/5,24
Pigment epithelial-differentiating factor	PEDF	gi|189778	131_18	9	46,471/5.84
Crystal Structure of PEDF	PEDF	gi|15988024	164_37	15	44,306/5.77
Serum amyloid P-component	SAMP	P02743	135_31	10	25,485/6.10
Serpin-Proteinase Complex	A1AT	gi|83764916	131_36	14	40,041/5.20
Serotransferrin	TF	P02787	390_50	40	79,294/6.81
Transthyretin	TTR	P02766	89_53	8	15,991/5.52

(a) AC represents accession numbers from the Swiss-Prot database. (b) Score is −10*Log(*p*), where *p* is the probability that the observed match is a random event; it is based on the Swiss-Prot database using the MASCOT searching program as MALDI-TOF MS data. (c) SC, sequence coverage.

**Table 3:** Sequence validation of selected proteins previously identified in post-ESWL urine samples by MS using LIFT technology.

ABBR. Name	Mw/ p*I* Theor.	PMF Score ^a^	Peptide Matched/ Peptide Searched	SC ^b^ %	Lift (MS_2_) Ion Parent Masses (*m*/*z*)	Score ^c^ Tof-Tof	Peptide Sequence
**MMP7**	29810/ 6.56	82	9/18	50	1784.9653 1492.8482 1139.6207	125	IVSYTRDLPHITVDR DLPHITVDRLVSK WTSKVVTYR
**DDAH2**	29915/ 5.66	89	7/19	36	2497.2438 459.7863	200	DFAVSTVPVSGPSHLRGLCGMGGI TVVAGSSDAAQKAVR
**CLU**	37002/ 5.74	110	12/28	42	2551.2517 1714.8468	167	FFTREPQDTYHYLPFSLPHR FMETVAEKALQEYR

^a^ PMF Score: Values are reported as Log_10_ (*p*), where *p* is the probability that the observed match is a random event, as calculated by the Swiss Prot database using the MASCOT search program; ^b^ SC, sequence coverage, is the ratio between the part of the sequence covered by the matched peptide and the full length of the protein sequence. ^c^ Score Tof-Tof: This value results from the combination of PMF and MS/MS of the matched peptide from ion parent fragments.

## Data Availability

All data have been included in the article.

## Supplementary Materials

The following supporting information can be downloaded at: https://www.mdpi.com/article/10.3390/biology14050482/s1, Figure S1: Flow chart for the creation of master gels related to pre-ESWL and post-ESWL conditions; Figure S2: Images for original Western blot gels. Table S1: List of genes for STRING analysis.
