# Supplementary material for: Significant Changes in Low-Abundance Protein Content Detected by Proteomic Analysis of Urine from Patients with Renal Stones After Extracorporeal Shock Wave Lithotripsy
Source: Biology (Basel). 2025 Apr 27;14(5):482. doi: 10.3390/biology14050482 (PMC12108638; doi:10.3390/biology14050482)
Supplement: Supplementary file 1 [file biology-14-00482-s001.zip › Table S1 .pdf]

Table S1: List of genes and related protein included for STRING analysis

1) 'A1AT':

SERPINA1 - Short peptide from AAT; Inhibitor of serine proteases. Its primary target is elastase, but it also has a moderate affinity for plasmin and thrombin. Irreversibly inhibits trypsin, chymotrypsin and plasminogen activator. The aberrant form inhibits insulin-induced NO synthesis in platelets, decreases coagulation time and has proteolytic activity against insulin and plasmin; Belongs to the serpin family. *[a.k.a. UPI00021CF3F2, CAA26677.1, NP_001121175.1, A1AT]*

2) 'ACTB':

ACTB - Actin, cytoplasmic 1, N-terminally processed; Actin is a highly conserved protein that polymerizes to produce filaments that form cross-linked networks in the cytoplasm of cells. Actin exists in both monomeric (G-actin) and polymeric (F-actin) forms, both forms playing key functions, such as cell motility and contraction. In addition to their role in the cytoplasmic cytoskeleton, G- and F-actin also localize in the nucleus, and regulate gene transcription and motility and repair of damaged DNA.

3) 'APOA4':

APOA4 - Apolipoprotein A-IV; May have a role in chylomicrons and VLDL secretion and catabolism. Required for efficient activation of lipoprotein lipase by ApoC-II; potent activator of LCAT. Apoa-IV is a major component of HDL and chylomicrons; Belongs to the apolipoprotein A1/A4/E family.

4) 'FCN3':

FCN3 - Ficolin-3; May function in innate immunity through activation of the lectin complement pathway. Calcium-dependent and GlcNAc-binding lectin. Has affinity with GalNAc, GlcNAc, D-fucose, as mono/oligosaccharide and lipopolysaccharides from S.typhimurium and S.minnesota.

5) 'APOE':

APOE - Apolipoprotein E; APOE is an apolipoprotein, a protein associating with lipid particles, that mainly functions in lipoprotein-mediated lipid transport between organs via the plasma and interstitial fluids. APOE is a core component of plasma lipoproteins and is involved in their production, conversion and clearance. Apoliproteins are amphipathic molecules that interact both with lipids of the lipoprotein particle core and the aqueous environment of the plasma. As such, APOE associates with chylomicrons, chylomicron remnants, very low density lipoproteins (VLDL) and intermediate density

6) 'DDAH2':

DDAH2 - N(G),N(G)-dimethylarginine dimethylaminohydrolase 2; Hydrolyzes N(G),N(G)-dimethyl-L-arginine (ADMA) and N(G)- monomethyl-L-arginine (MMA) which act as inhibitors of NOS. Has therefore a role in the regulation of nitric oxide generation.

7) 'ACTG1':

ACTG1 - Actin, cytoplasmic 2, N-terminally processed; Actins are highly conserved proteins that are involved in various types of cell motility and are ubiquitously expressed in all eukaryotic cells.

8) 'MMP7':

MMP7 - Matrilysin; Degrades casein, gelatins of types I, III, IV, and V, and fibronectin. Activates procollagenase.
TIMP1 - Metalloproteinase inhibitor 1; Metalloproteinase inhibitor that functions by forming one to one complexes with target metalloproteinases, such as collagenases, and irreversibly inactivates them by binding to their catalytic zinc cofactor. Acts on MMP1, MMP2, MMP3, MMP7, MMP8, MMP9, MMP10, MMP11, MMP12, MMP13 and MMP16. Does not act on MMP14. Also functions as a growth factor that regulates cell differentiation, migration and cell death and activates cellular signaling cascades via CD63 and ITGB1. Plays a role in integrin signaling. Mediates erythropoiesis in vitro; but, unlike IL3, it [...] *[a.k.a. TIMP, Q6FGX5, TIMP metallopeptidase inhibitor 1]*

9) 'Hem2':

NCKAP1 - Nck-associated protein 1; Part of the WAVE complex that regulates lamellipodia formation. The WAVE complex regulates actin filament reorganization via its interaction with the Arp2/3 complex. Actin remodeling activity is regulated by RAC1. As component of the WAVE1 complex, required for BDNF-NTRK2 endocytic trafficking and signaling from early endosomes. *[a.k.a. ENSP00000354251, R-HSA-162582, Q9Y2A7, HEM2]*

10) 'UBQLN4':

UBQLN4 - Ubiquilin-4; Regulator of protein degradation that mediates the proteasomal targeting of misfolded, mislocalized or accumulated proteins. Acts by binding polyubiquitin chains of target proteins via its UBA domain and by interacting with subunits of the proteasome via its ubiquitin-like domain. Key regulator of DNA repair that represses homologous recombination repair: in response to DNA damage, recruited to sites of DNA damage following phosphorylation by ATM and acts by binding and removing ubiquitinated MRE11 from damaged chromatin, leading to MRE11 degradation by the proteasome. MRE [...]

11) 'C3':
C3 - Complement C3c alpha' chain fragment 1; C3 plays a central role in the activation of the complement system. Its processing by C3 convertase is the central reaction in both classical and alternative complement pathways. After activation C3b can bind covalently, via its reactive thioester, to cell surface carbohydrates or immune aggregates. [C3-beta-c]: Acts as a chemoattractant for neutrophils in chronic inflammation.
CFP - Properdin; A positive regulator of the alternate pathway (AP) of complement. It binds to and stabilizes the C3- and C5-convertase enzyme complexes. Inhibits CFI-CFH mediated degradation of Complement C3 beta chain (C3b). *[a.k.a. ENSP00000366204, XP_016885064.1, 5199]*
12) 'HBB':
HBB - Hemoglobin subunit beta; Involved in oxygen transport from the lung to the various peripheral tissues. [Spinorphin]: functions as an endogenous inhibitor of enkephalin-degrading enzymes such as DPP3, and as a selective antagonist of the P2RX3 receptor which is involved in pain signaling, these properties implicate it as a regulator of pain and inflammation; Belongs to the globin family.
13) 'ATX3':
ATXN3 - Ataxin-3; Deubiquitinating enzyme involved in protein homeostasis maintenance, transcription, cytoskeleton regulation, myogenesis and degradation of misfolded chaperone substrates. Binds long polyubiquitin chains and trims them, while it has weak or no activity against chains of 4 or less ubiquitins. Involved in degradation of misfolded chaperone substrates via its interaction with STUB1/CHIP: recruited to monoubiquitinated STUB1/CHIP, and restricts the length of ubiquitin chain attached to STUB1/CHIP substrates and preventing further chain extension (By similarity).
14) 'CLU':
CLU - Clusterin alpha chain; [Isoform 1]: Functions as extracellular chaperone that prevents aggregation of non native proteins. Prevents stress-induced aggregation of blood plasma proteins. Inhibits formation of amyloid fibrils by APP, APOC2, B2M, CALCA, CSN3, SNCA and aggregation-prone LYZ variants (in vitro). Does not require ATP. Maintains partially unfolded proteins in a state appropriate for subsequent refolding by other chaperones, such as HSPA8/HSC70. Does not refold proteins by itself. Binding to cell surface receptors triggers internalization of the chaperone-client complex
15) 'APO E':
APOE - Apolipoprotein E; APOE is an apolipoprotein, a protein associating with lipid particles, that mainly functions in lipoprotein-mediated lipid transport between organs via the plasma and interstitial fluids. APOE is a core component of plasma lipoproteins and is involved in their production, conversion and clearance. Apoliproteins are amphipathic molecules that interact both with lipids of the lipoprotein particle core and the aqueous environment of the plasma. As such, APOE associates with chylomicrons, chylomicron remnants, very low density lipoproteins (VLDL) and intermediate density
16) 'DYNC1LI1':
DYNC1LI1 - Cytoplasmic dynein 1 light intermediate chain 1; Acts as one of several non-catalytic accessory components of the cytoplasmic dynein 1 complex that are thought to be involved in linking dynein to cargos and to adapter proteins that regulate dynein function. Cytoplasmic dynein 1 acts as a motor for the intracellular retrograde motility of vesicles and organelles along microtubules. May play a role in binding dynein to membranous organelles or chromosomes. Probably involved in the microtubule-dependent transport of pericentrin. Is required for progress through the spindle assembly
17) 'FGG':
FGG - Fibrinogen gamma chain; Together with fibrinogen alpha (FGA) and fibrinogen beta (FGB), polymerizes to form an insoluble fibrin matrix. Has a major function in hemostasis as one of the primary components of blood clots. In addition, functions during the early stages of wound repair to stabilize the lesion and guide cell migration during re- epithelialization. Was originally thought to be essential for platelet aggregation, based on in vitro studies using anticoagulated blood. However, subsequent studies have shown that it is not absolutely required for thrombus formation in vivo.
18) 'TPP1':
TPP1 - Tripeptidyl-peptidase 1; Lysosomal serine protease with tripeptidyl-peptidase I activity. May act as a non-specific lysosomal peptidase which generates tripeptides from the breakdown products produced by lysosomal proteinases. Requires substrates with an unsubstituted N-terminus.
19) 'TTR':
TTR - Transthyretin; Thyroid hormone-binding protein. Probably transports thyroxine from the bloodstream to the brain.
ONECUT1 - Hepatocyte nuclear factor 6; Transcriptional activator. Binds the consensus sequence 5'-DHWATTGAYTWWD-3' on a variety of gene promoters such as those of HNF3B and TTR. Important for liver genes transcription; Belongs to the CUT homeobox family.

20) 'A1BG':
A1BG - Alpha-1B-glycoprotein; alpha-1-B glycoprotein.
21) 'AMBP':
AMBP - Inter-alpha-trypsin inhibitor light chain; Inter-alpha-trypsin inhibitor inhibits trypsin, plasmin, and lysosomal granulocytic elastase. Inhibits calcium oxalate crystallization; In the N-terminal section; belongs to the calycin superfamily. Lipocalin family.
22) 'SERPINC1':
SERPINC1 - Antithrombin-III; Most important serine protease inhibitor in plasma that regulates the blood coagulation cascade. AT-III inhibits thrombin, matriptase-3/TMPRSS7, as well as factors IXa, Xa and XIa. Its inhibitory activity is greatly enhanced in the presence of heparin.
23) 'APOA1':
APOA1 - Truncated apolipoprotein A-I; Participates in the reverse transport of cholesterol from tissues to the liver for excretion by promoting cholesterol efflux from tissues and by acting as a cofactor for the lecithin cholesterol acyltransferase (LCAT). As part of the SPAP complex, activates spermatozoa motility.
24) 'ENO1':
ENO1 - Alpha-enolase; Glycolytic enzyme the catalyzes the conversion of 2- phosphoglycerate to phosphoenolpyruvate. In addition to glycolysis, involved in various processes such as growth control, hypoxia tolerance and allergic responses. May also function in the intravascular and pericellular fibrinolytic system due to its ability to serve as a receptor and activator of plasminogen on the cell surface of several cell-types such as leukocytes and neurons. Stimulates immunoglobulin production. Belongs to the enolase family.
25) 'CP':
CP - Ceruloplasmin; Ceruloplasmin is a blue, copper-binding (6-7 atoms per molecule) glycoprotein. It has ferroxidase activity oxidizing $Fe(2+)$ to $Fe(3+)$ without releasing radical oxygen species. It is involved in iron transport across the cell membrane. Provides $Cu(2+)$ ions for the ascorbate-mediated deaminase degradation of the heparan sulfate chains of GPC1. May also play a role in fetal lung development or pulmonary antioxidant defense (By similarity).
26) 'C9':
C9 - Complement component C9a; Constituent of the membrane attack complex (MAC) that plays a key role in the innate and adaptive immune response by forming pores in the plasma membrane of target cells. C9 is the pore-forming subunit of the MAC.
27) 'CFB':
CFB - Complement factor B Ba fragment; Factor B which is part of the alternate pathway of the complement system is cleaved by factor D into 2 fragments: Ba and Bb. Bb, a serine protease, then combines with complement factor 3b to generate the C3 or C5 convertase. It has also been implicated in proliferation and differentiation of preactivated B-lymphocytes, rapid spreading of peripheral blood monocytes, stimulation of lymphocyte blastogenesis and lysis of erythrocytes. Ba inhibits the proliferation of preactivated B-lymphocytes.
28) 'C3':
C3 - Complement C3c alpha' chain fragment 1; C3 plays a central role in the activation of the complement system. Its processing by C3 convertase is the central reaction in both classical and alternative complement pathways. After activation C3b can bind covalently, via its reactive thioester, to cell surface carbohydrates or immune aggregates. [C3-beta-c]: Acts as a chemoattractant for neutrophils in chronic inflammation.
29) 'CFH':
CFH - Complement factor H; Glycoprotein that plays an essential role in maintaining a well-balanced immune response by modulating complement activation. Acts as a soluble inhibitor of complement, where its binding to self markers such as glycan structures prevents complement activation and amplification on cell surfaces. Accelerates the decay of the complement alternative pathway (AP) C3 convertase C3bBb, thus preventing local formation of more C3b, the central player of the complement amplification loop. As a cofactor of the serine protease factor I, CFH also regulates proteolytic degradation
30) 'FCN3':
FCN3 - Ficolin-3; May function in innate immunity through activation of the lectin complement pathway. Calcium-dependent and GlcNAc-binding lectin. Has affinity with GalNAc, GlcNAc, D-fucose, as mono/oligosaccharide and lipopolysaccharides from S.typhimurium and S.minnesota.
31) 'FBLN1':
FBLN1 - Fibulin-1; Incorporated into fibronectin-containing matrix fibers. May play a role in cell adhesion and migration along protein fibers within the extracellular matrix (ECM). Could be important for certain developmental processes and contribute to the supramolecular organization of ECM architecture, in particular to those of basement membranes. Has been implicated in a role in cellular transformation and tumor invasion,

it appears to be a tumor suppressor. May play a role in haemostasis and thrombosis owing to its ability to bind fibrinogen and incorporate into clots.

32) 'QPCT':

QPCT - Glutaminyl-peptide cyclotransferase; Responsible for the biosynthesis of pyroglutamyl peptides. Has a bias against acidic and tryptophan residues adjacent to the N- terminal glutaminyl residue and a lack of importance of chain length after the second residue. Also catalyzes N-terminal pyroglutamate formation. In vitro, catalyzes pyroglutamate formation of N-terminally truncated form of APP amyloid-beta peptides [Glu-3]-amyloid-beta. May be involved in the N-terminal pyroglutamate formation of several amyloid-related plaque-forming peptides.

33) 'HSPB1':

HSPB1 - Heat shock protein beta-1; Small heat shock protein which functions as a molecular chaperone probably maintaining denatured proteins in a folding- competent state. Plays a role in stress resistance and actin organization. Through its molecular chaperone activity may regulate numerous biological processes including the phosphorylation and the axonal transport of neurofilament proteins.

34) 'KRT19':

KRT19 - Keratin, type I cytoskeletal 19; Involved in the organization of myofibers. Together with KRT8, helps to link the contractile apparatus to dystrophin at the costameres of striated muscle; Belongs to the intermediate filament family.

35) 'Map19':

MASP2 - Mannan-binding lectin serine protease 2 A chain; Serum protease that plays an important role in the activation of the complement system via mannose-binding lectin. After activation by auto-catalytic cleavage it cleaves C2 and C4, leading to their activation and to the formation of C3 convertase.

36) 'PNMA6C':

PNMA6A - Paraneoplastic antigen-like protein 6A; PNMA family member 6A; Belongs to the PNMA family.

37) 'PEDF':

SERPINF1 - Pigment epithelium-derived factor; Neurotrophic protein; induces extensive neuronal differentiation in retinoblastoma cells. Potent inhibitor of angiogenesis. As it does not undergo the S (stressed) to R (relaxed) conformational transition characteristic of active serpins, it exhibits no serine protease inhibitory activity.

38) 'APCS':

APCS - Serum amyloid P-component(1-203); Can interact with DNA and histones and may scavenge nuclear material released from damaged circulating cells. May also function as a calcium-dependent lectin; Belongs to the pentraxin family.

39) 'TF':

TF - Serotransferrin; Transferrins are iron binding transport proteins which can bind two $Fe(3+)$ ions in association with the binding of an anion, usually bicarbonate. It is responsible for the transport of iron from sites of absorption and heme degradation to those of storage and utilization. Serum transferrin may also have a further role in stimulating cell proliferation.

40) 'TTR':

TTR - Transthyretin; Thyroid hormone-binding protein. Probably transports thyroxine from the bloodstream to the brain.

41

41) 'L-lactate dehydrogenase':

LDHAL6B - Lactate dehydrogenase A like 6B. ***[a.k.a. AAH22034.1, Q9BYZ2, 1.1.1.27, L-lactate dehydrogenase]***
