# Supplementary figures and images for: Significant Changes in Low-Abundance Protein Content Detected by Proteomic Analysis of Urine from Patients with Renal Stones After Extracorporeal Shock Wave Lithotripsy
Source: Biology (Basel). 2025 Apr 27;14(5):482. doi: 10.3390/biology14050482 (PMC12108638; doi:10.3390/biology14050482)

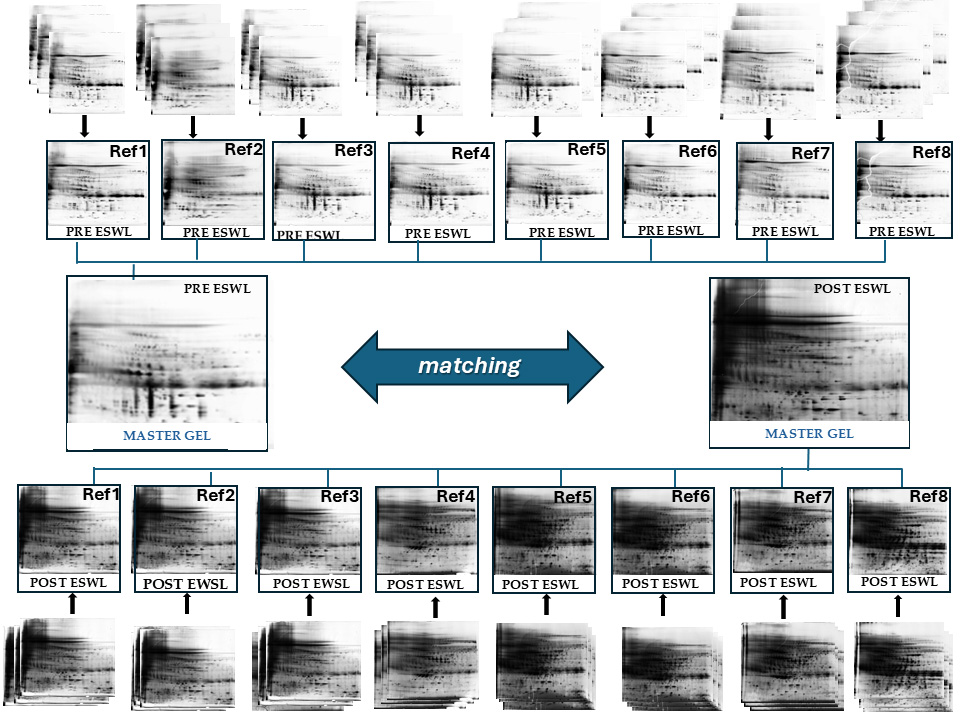

Supplement: Supplementary file 1 [file biology-14-00482-s001.zip › Figure S1.jpg]

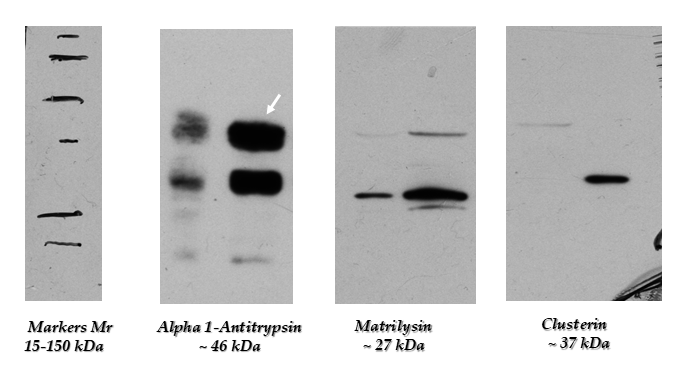

Supplement: Supplementary file 1 [file biology-14-00482-s001.zip › Figure S2. ORIGINAL WB.tif]
